# Supplementary material for: Whole-genome sequencing of acral melanoma reveals genomic complexity and diversity
Source: Nat Commun. 2020 Oct 16;11:5259. doi: 10.1038/s41467-020-18988-3 (PMC7567804; doi:10.1038/s41467-020-18988-3)
Supplement: Supplementary file 1 — Supplementary Information [file 41467_2020_18988_MOESM1_ESM.pdf]

SUPPLEMENTARY INFORMATION

Whole genome sequencing of acral melanoma reveals genomic complexity and diversity, Newell *et al*

Supplementary Figure 1: Associations of clinicopathological features.
Supplementary Figure 2: Rearrangement and CNV signature probabilities and cosine similarity.
Supplementary Figure 3: Associations with aneuploidy
Supplementary Figure 4: Associations with localized complex genomic rearrangements
Supplementary Figure 5: Examples of localized complex events on recurrent chromosomes
Supplementary Figure 6: Recurrent linking translocations
Supplementary Figure 7: Significantly mutated genes
Supplementary Figure 8: GISTIC analysis in tumor subgroups
Supplementary Figure 9: Regions of recurrent rearrangement breakpoints in tumor subgroups
Supplementary Figure 10: Correlation of gene mutations with expression
Supplementary Figure 11: Survival in primary acral melanomas with and without *PTEN* aberrations
Supplementary Figure 12: Neoantigens and the tumor microenvironment

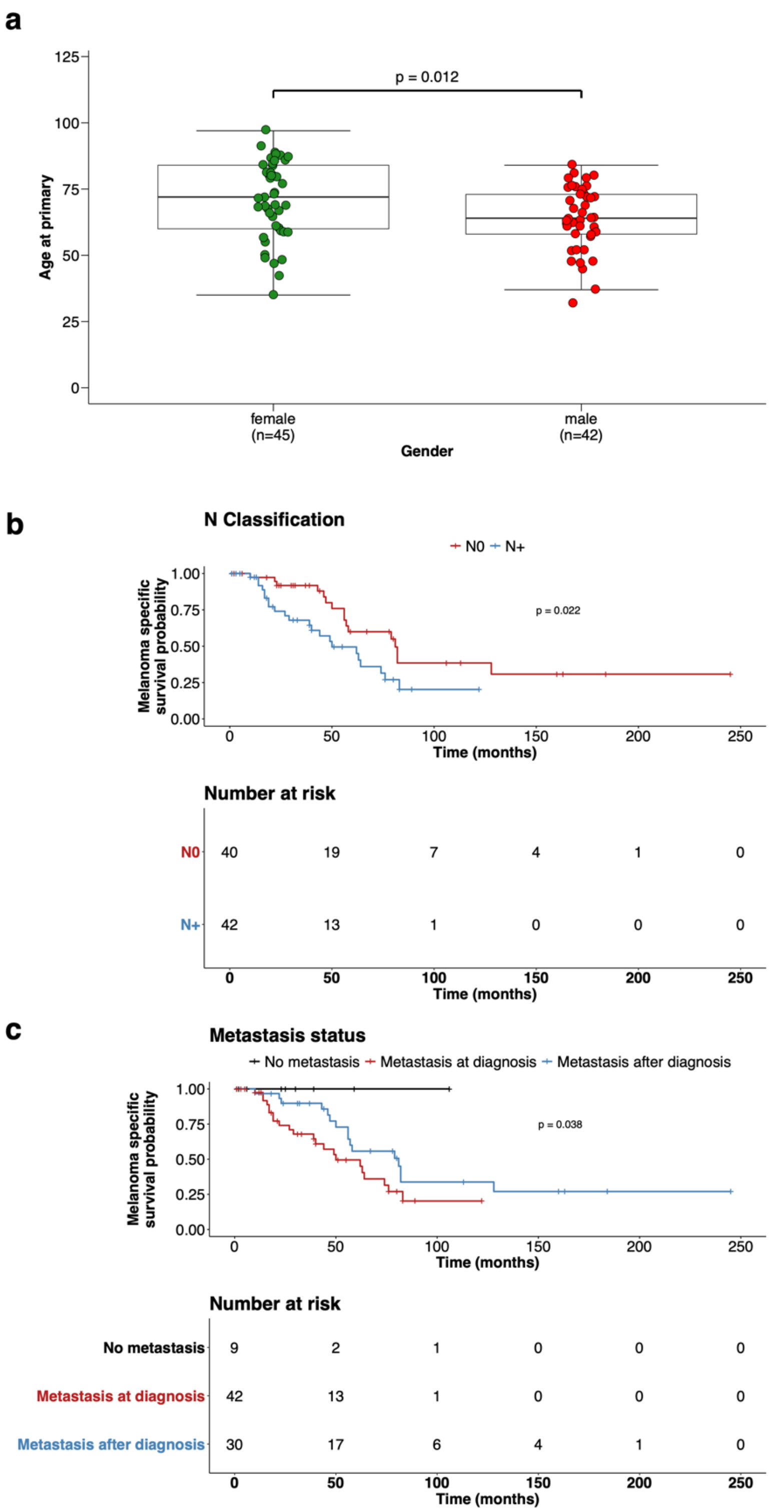

Supplementary Figure 1: Associations of clinicopathological features. a, Box plot of age at diagnosis of primary in males and females (Mann-Whitney U test). The box boundaries show the first to third quartiles, the median is the centre line and the whiskers represent 1.5 times the inter-quartile range. **b** and **c**, Kaplan-Meier curves showing melanoma-specific survival using a log-rank test. **b**, N classification. **c,** Metastasis status comparing patients with no metastasis, metastasis present when diagnosed and metastasis after diagnosis.

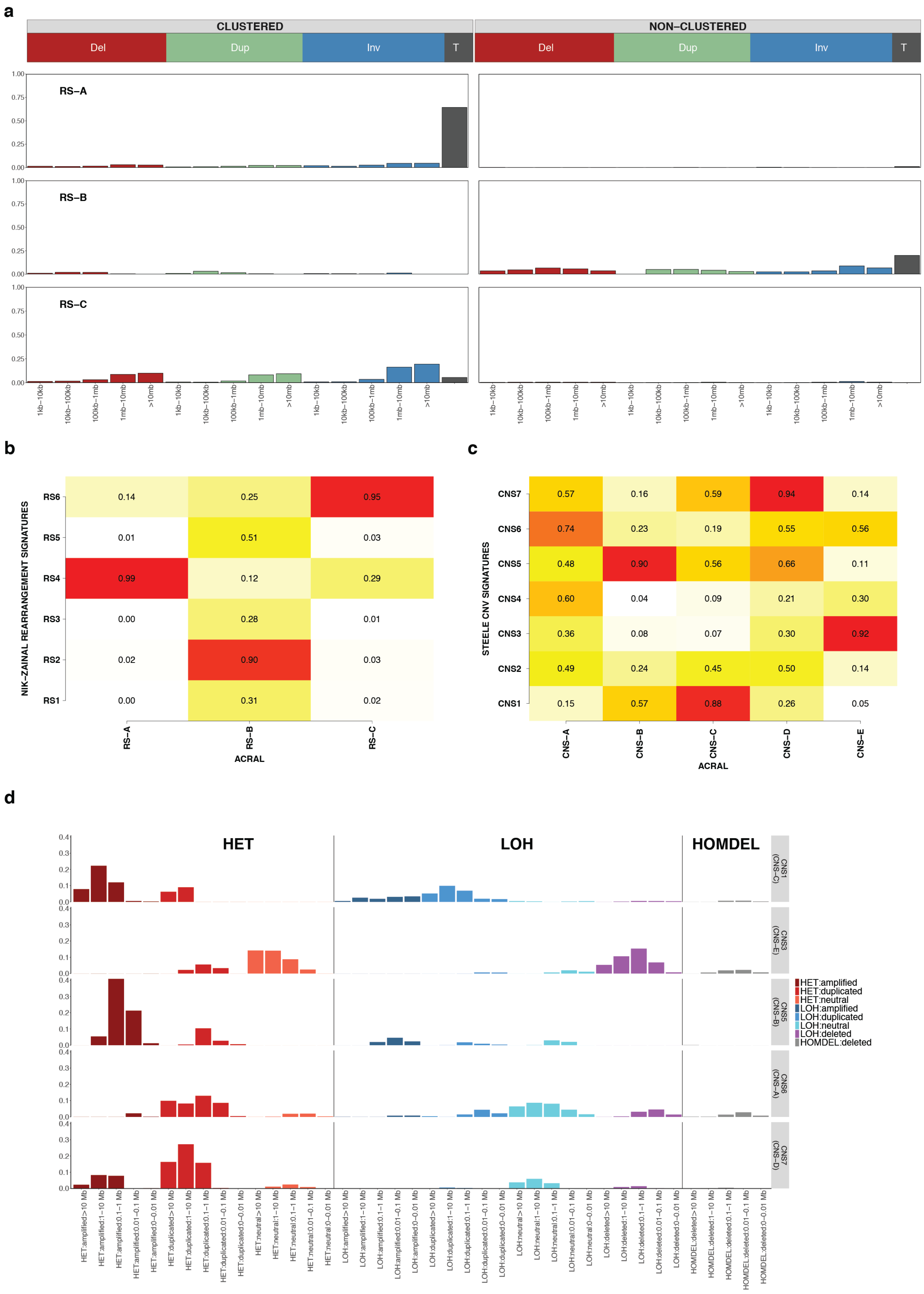
a
CLUSTERED
NON–CLUSTERED
Del
Dup
Inv
T
RS–A
RS–B
RS–C
1.00
0.75
0.50
0.25
0.00
1kb–10kb
10kb–100kb
100kb–1mb
1mb–10mb
>10mb
b
NIK-ZAINAL REARRANGEMENT SIGNATURES
RS6 0.14 0.25 0.95
RS5 0.01 0.51 0.03
RS4 0.99 0.12 0.29
RS3 0.00 0.28 0.01
RS2 0.02 0.90 0.03
RS1 0.00 0.31 0.02
RS–A RS–B RS–C
ACRAL
c
STEELE CNV SIGNATURES
CNS7 0.57 0.16 0.59 0.94 0.14
CNS6 0.74 0.23 0.19 0.55 0.56
CNS5 0.48 0.90 0.56 0.66 0.11
CNS4 0.60 0.04 0.09 0.21 0.30
CNS3 0.36 0.08 0.07 0.30 0.92
CNS2 0.49 0.24 0.45 0.50 0.14
CNS1 0.15 0.57 0.88 0.26 0.05
CNS–A CNS–B CNS–C CNS–D CNS–E
ACRAL
d
HET
LOH
HOMDEL
CNS1 (CNS–C)
CNS3 (CNS–E)
CNS5 (CNS–B)
CNS6 (CNS–A)
CNS7 (CNS–D)
0.4
0.3
0.2
0.1
0.0
HET:amplified
HET:duplicated
HET:neutral
LOH:amplified
LOH:duplicated
LOH:neutral
LOH:deleted
HOMDEL:deleted
HET:amplified:>10 Mb
HET:amplified:1–10 Mb
HET:amplified:0.1–1 Mb
HET:amplified:0.01–0.1 Mb
HET:amplified:0–0.01 Mb
HET:duplicated:>10 Mb
HET:duplicated:1–10 Mb
HET:duplicated:0.1–1 Mb
HET:duplicated:0.01–0.1 Mb
HET:duplicated:0–0.01 Mb
HET:neutral:>10 Mb
HET:neutral:1–10 Mb
HET:neutral:0.1–1 Mb
HET:neutral:0.01–0.1 Mb
HET:neutral:0–0.01 Mb
LOH:amplified:>10 Mb
LOH:amplified:1–10 Mb
LOH:amplified:0.1–1 Mb
LOH:amplified:0.01–0.1 Mb
LOH:amplified:0–0.01 Mb
LOH:duplicated:>10 Mb
LOH:duplicated:1–10 Mb
LOH:duplicated:0.1–1 Mb
LOH:duplicated:0.01–0.1 Mb
LOH:duplicated:0–0.01 Mb
LOH:neutral:>10 Mb
LOH:neutral:1–10 Mb
LOH:neutral:0.1–1 Mb
LOH:neutral:0.01–0.1 Mb
LOH:neutral:0–0.01 Mb
LOH:deleted:>10 Mb
LOH:deleted:1–10 Mb
LOH:deleted:0.1–1 Mb
LOH:deleted:0.01–0.1 Mb
LOH:deleted:0–0.01 Mb
HOMDEL:deleted:>10 Mb
HOMDEL:deleted:1–10 Mb
HOMDEL:deleted:0.1–1 Mb
HOMDEL:deleted:0.01–0.1 Mb
HOMDEL:deleted:0–0.01 Mb

Supplementary Figure 2: Rearrangement and CNV signature probabilities and cosine similarity. a, Three rearrangement signatures were extracted using non-negative matrix factorization (NMF) and are grouped by clustered and non-clustered deletions (Del), duplications (Dup), Inversions (Inv) of different sizes and translocations (T). The y axis shows the probability of each category type. **b,** Cosine similarity of rearrangement signatures with published breast cancer signatures. **c**, Cosine similarity of CNV signatures with published sarcoma CNV signatures. **d**, Five CNV signatures were extracted using NMF and are grouped into 40 categories. HET, heterozygous; LOH, loss of heterozygosity; HOMDEL, homozygous deletion. The y axis shows the probability of each category type. CNS1 is a signature of amplified LOH and CNS3 occurs in samples with a large proportion of unaltered segments with some small amplifications and large deletions. CNS5 is associated with amplified regions with retention of heterozygosity and CNS6 is a signature of large heterozygous duplicated neutral LOH segments. CNS7 is a signature with retention of heterozygosity with low level gains.

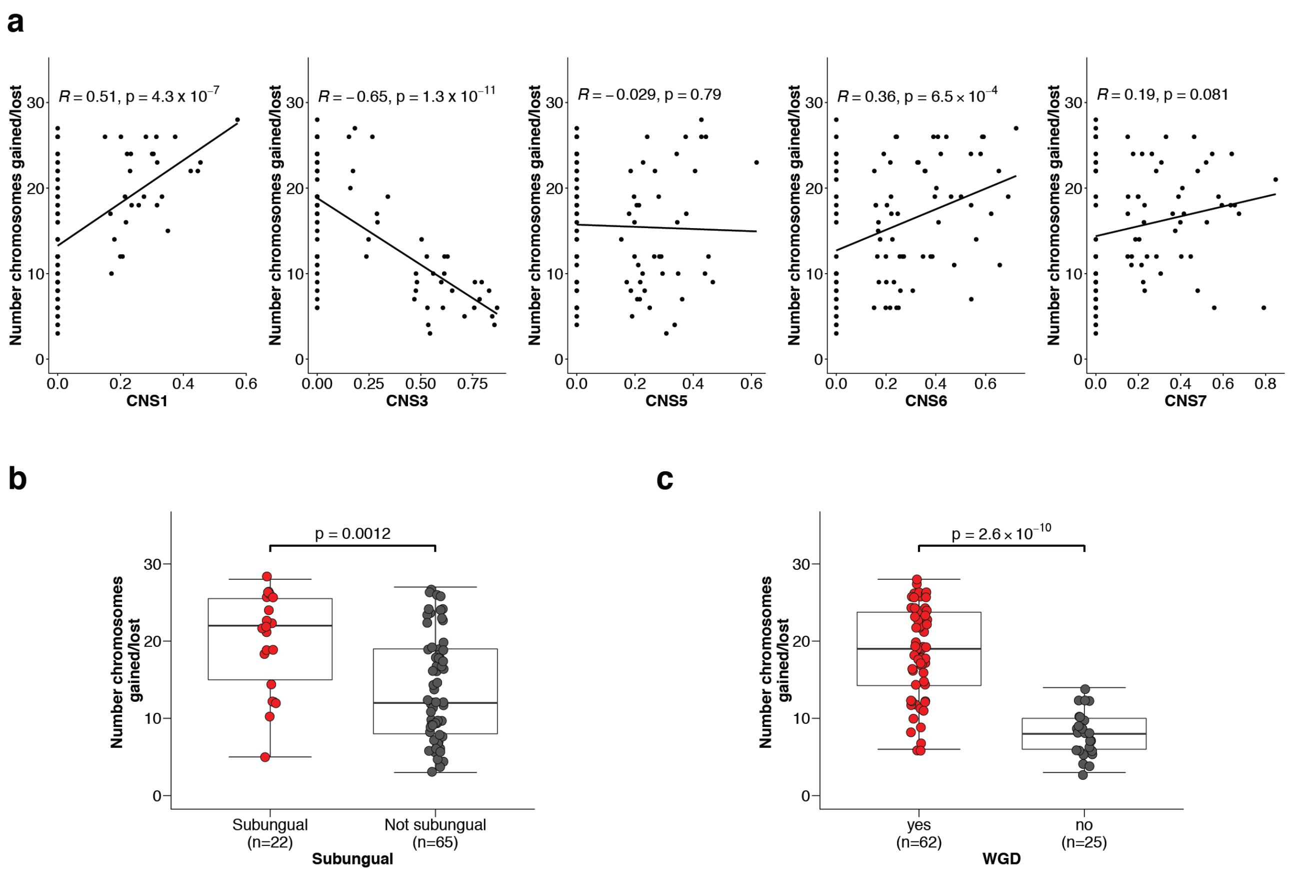

Supplementary Figure 3: Associations with aneuploidy. a, Pearson's correlation of CNV signatures CNS1, CNS3, CNS5, CNS6, CNS7 with the number of chromosome arms gained and lost. **b,** Box plot of aneuploidy in tumors that are subungual compared with tumors from other sites (Mann-Whitney U test). **c,** Box plot of aneuploidy in tumors that are diploid or have undergone whole genome duplication (Mann-Whitney U test). In each box plot, the box boundaries show the first to third quartiles, the median is the centre line and the whiskers represent 1.5 times the inter-quartile range.

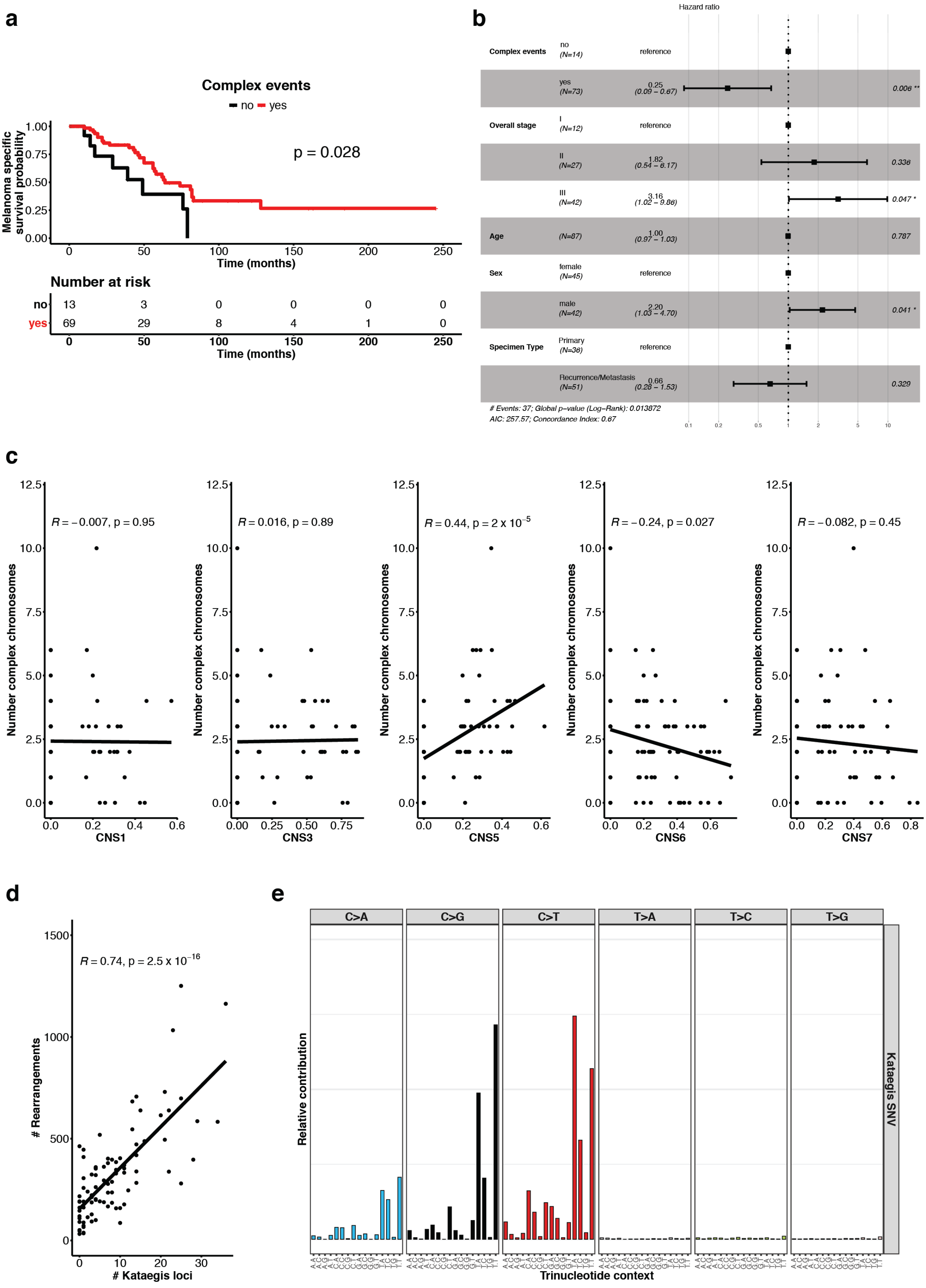
a
Complex events
no yes
Melanoma specific survival probability
1.00
0.75
0.50
0.25
0.00
p = 0.028
0 50 100 150 200 250
Time (months)
Number at risk
no 13 3 0 0 0 0
yes 69 29 8 4 1 0
0 50 100 150 200 250
Time (months)
b
Hazard ratio
Complex events no (N=14) reference
yes (N=73) 0.25 (0.09 – 0.67) 0.006 **
Overall stage I (N=12) reference
II (N=27) 1.82 (0.54 – 6.17) 0.336
III (N=42) 3.16 (1.02 – 9.86) 0.047 *
Age (N=87) 1.00 (0.97 – 1.03) 0.787
Sex female (N=45) reference
male (N=42) 2.20 (1.03 – 4.70) 0.041 *
Specimen Type Primary (N=38) reference
Recurrence/Metastasis (N=51) 0.66 (0.28 – 1.53) 0.329
Events: 37; Global p-value (Log-Rank): 0.013872
AIC: 257.57; Concordance Index: 0.67
0.1 0.2 0.5 1 2 5 10
c
Number complex chromosomes
R = −0.007, p = 0.95
CNS1
R = 0.016, p = 0.89
CNS3
R = 0.44, p = 2 x 10−5
CNS5
R = −0.24, p = 0.027
CNS6
R = −0.082, p = 0.45
CNS7
d
R = 0.74, p = 2.5 x 10−16
# Rearrangements
# Kataegis loci
e
C>A C>G C>T T>A T>C T>G
Relative contribution
Kataegis SNV
Trinucleotide context

Supplementary Figure 4: Associations with localized complex genomic rearrangements
a, Kaplan-Meier plot of melanoma-specific survival with log-rank test in tumors with or without localized complex rearrangements. **b**, Forest plot for a multivariable Cox survival model based on presence of localized complex rearrangements, overall stage, patient age at diagnosis of primary, sex and specimen type (primary or recurrence/metastasis). **c,** Pearson's correlation of CNV signatures CNS1, CNS3, CNS5, CNS6, CNS7 with the number of chromosomes per tumor with localised complex rearrangements **d**, Pearson's correlation of the number of kataegis loci per tumor with structural rearrangement count. **e**, Trinucleotide context of SNVs that fall within identified kataegis loci tumors showing that there is a similarity to APOBEC signatures SBS2 and SBS13.

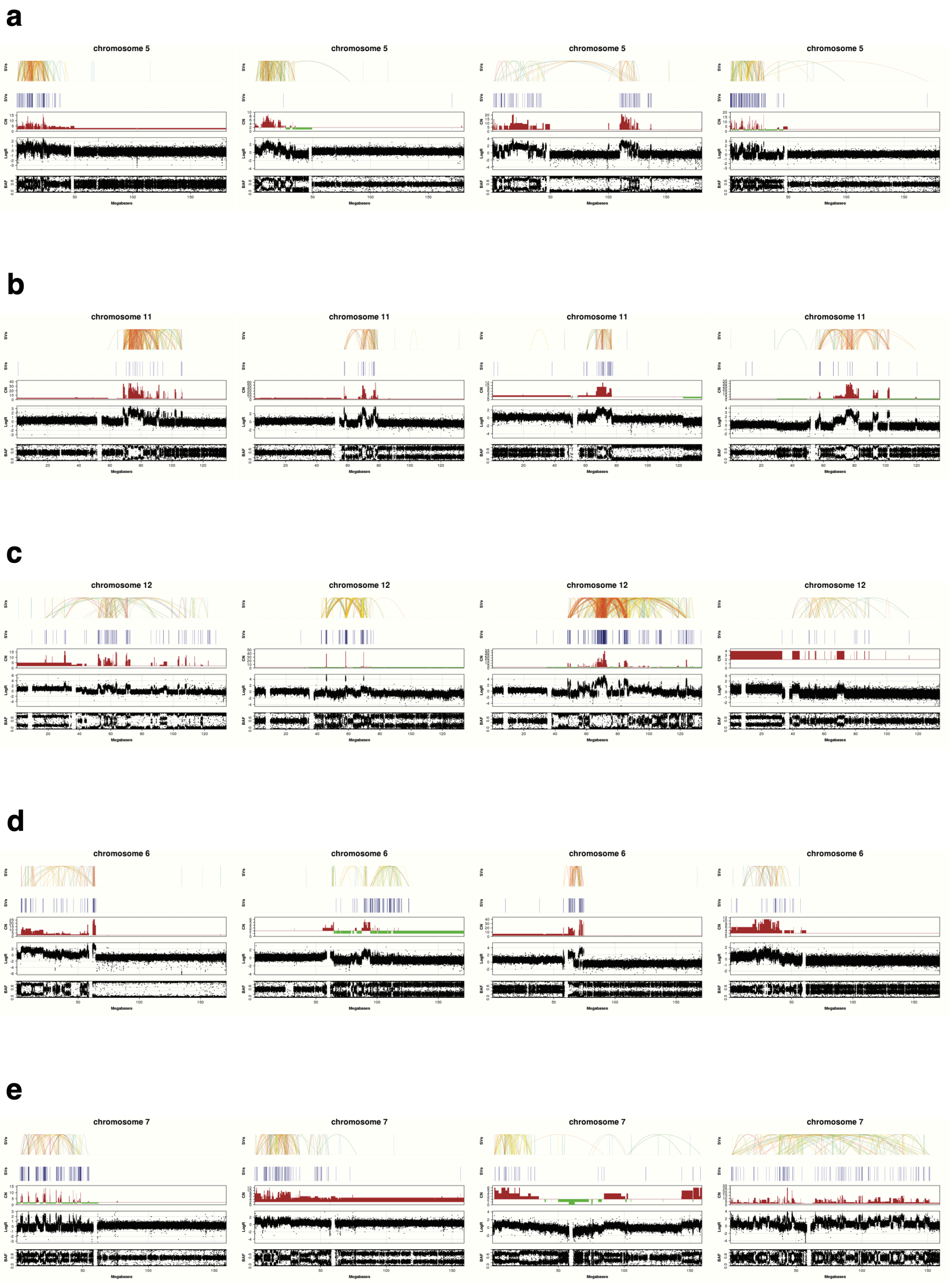

Supplementary Figure 5: Examples of localized complex events on recurrent chromosomes. **a,** chromosome 5**. b,** chromosome 11**. c,** chromosome 12**. d,** chromosome 6**. e,** chromosome 7**.**

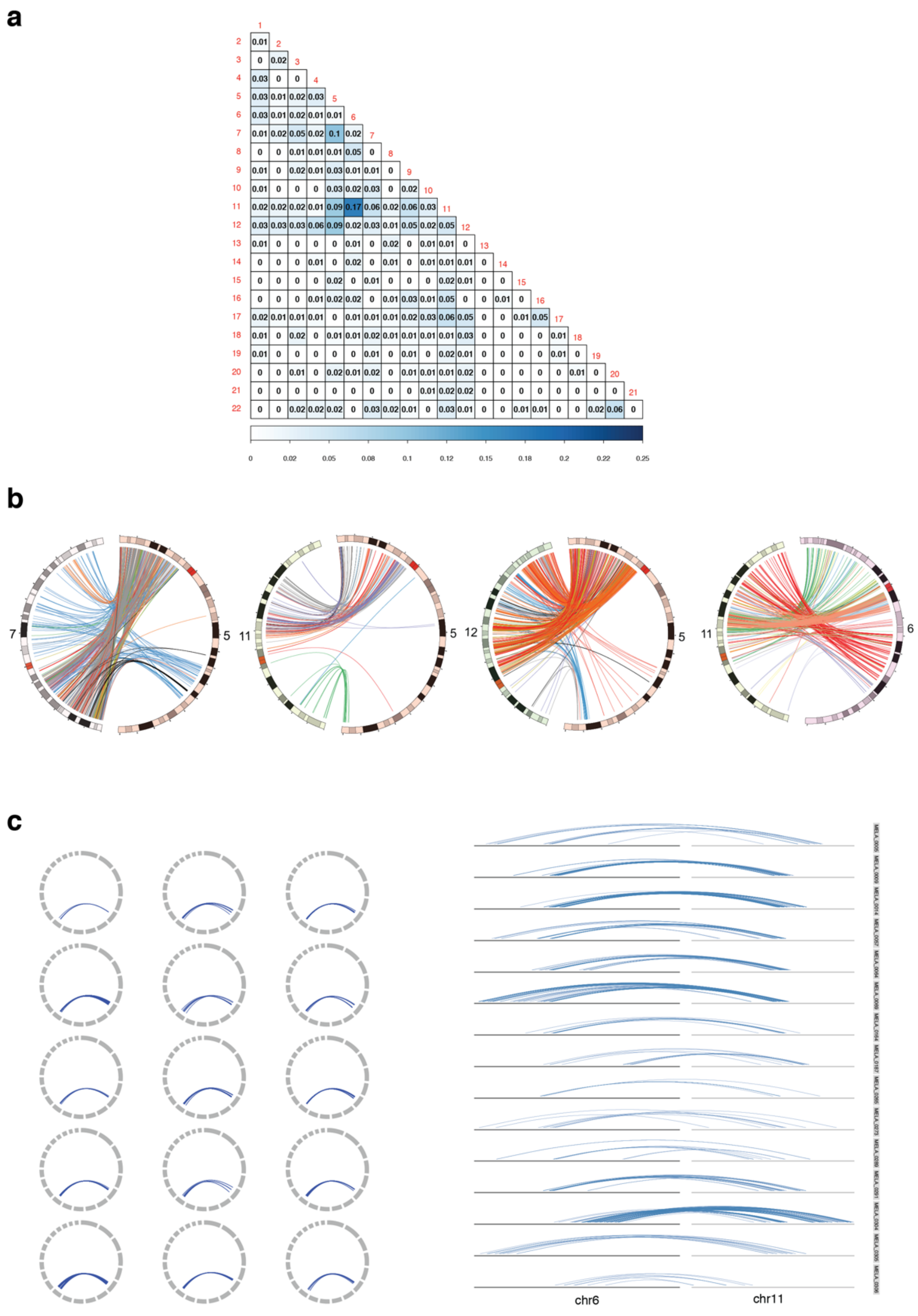

Supplementary Figure 6: Recurrent linking translocations. a, Proportion of tumors with 5 or more linking translocations between chromosomes. **b**, Circos plots showing translocations between (left to right) chromosomes 5 and 7; chromosomes 5 and 11; chromosomes 5 and 12 and chromosomes 6 and 11 for tumors with 5 or more linking translocations. Each colour represents the translocations for one tumor. **c**, Circos plots for chromosome 6 to chromosome 11 translocations for each tumor (left) and the location on the chromosome of each linking translocation.

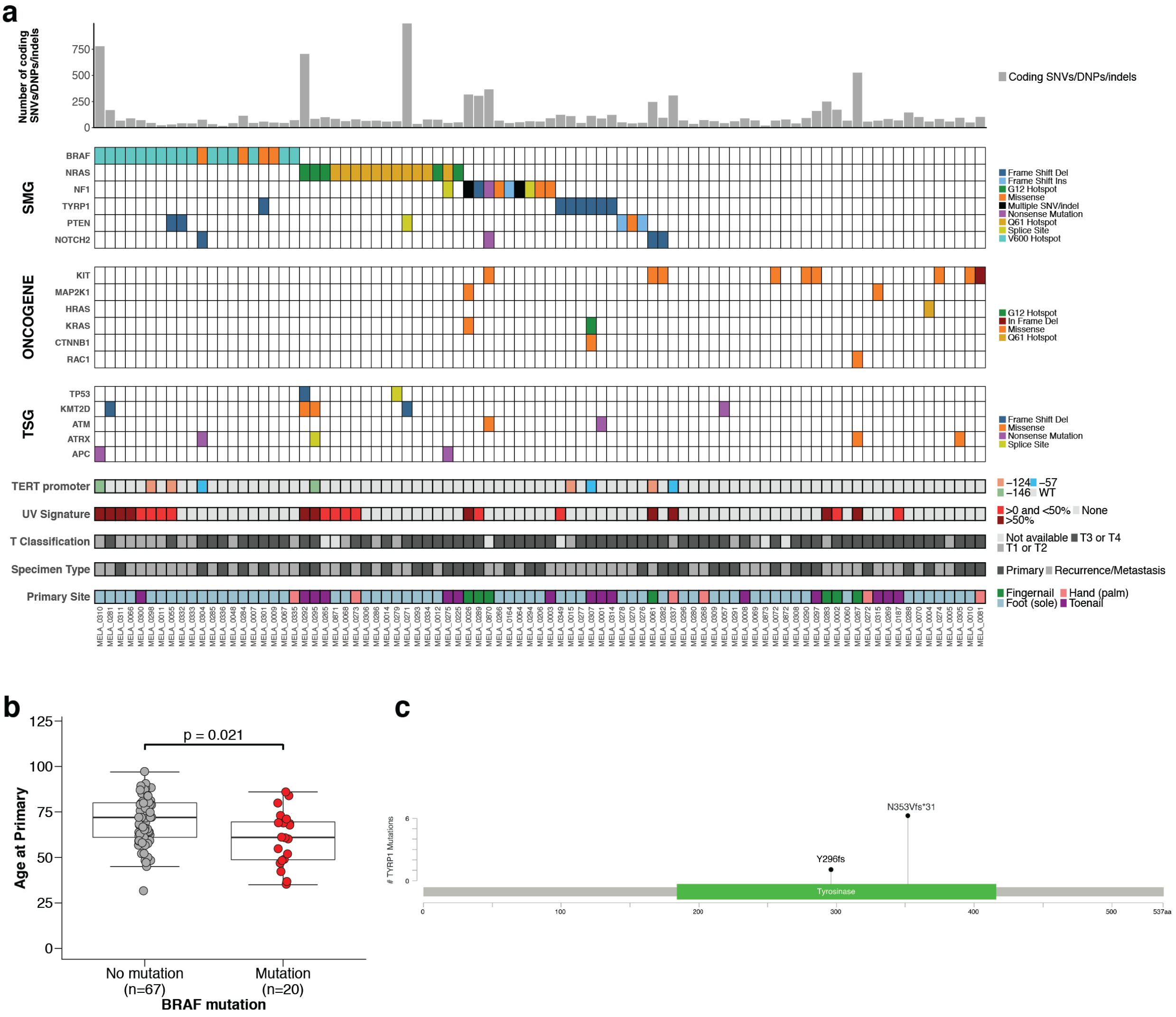

Supplementary Figure 7: Significantly mutated genes. a, Oncoplot showing from top to bottom: number of coding SNV/indels; mutations in significantly mutated genes; mutations in oncogenes; mutations in tumor suppressor genes (TSG); *TERT* promoter mutations; presence of UVR signature; T classification; specimen type and primary site per sample. The barchart for the number of coding SNV/indels in the upper panel represents the counts from n=87 tumors, where each bar represents the counts for a single tumor. For oncogenes, nine tumors had a *KIT* mutation, two tumors had a hotspot mutation in *MAP2K1* (2 at p.C21S) and two with *KRAS* mutations (1 at the Q61 hotspot and 1 at p.D33E) and single mutations occurred in *HRAS* (G12 hotspot), *RAC1* (P29S) and *CTNNB1* (P37C) were also identified. Mutations, including putatively protein truncating loss of function (LoF) mutations, also occurred in tumor suppressors such *TP53*, *KMT2D*, *ATRX*, *ATM* and *APC*. **b,** Box plot of age at diagnosis of the primary lesion in the presence or absence of *BRAF* mutations (Mann-Whitney U test). In each box plot, the box boundaries show the first to third quartiles, the median is the centre line and the whiskers represent 1.5 times the inter-quartile range. **c,** Position of mutations in TYRP1 protein. Mutations are coloured black (frame shift) and the tyrosine protein domain is coloured green.

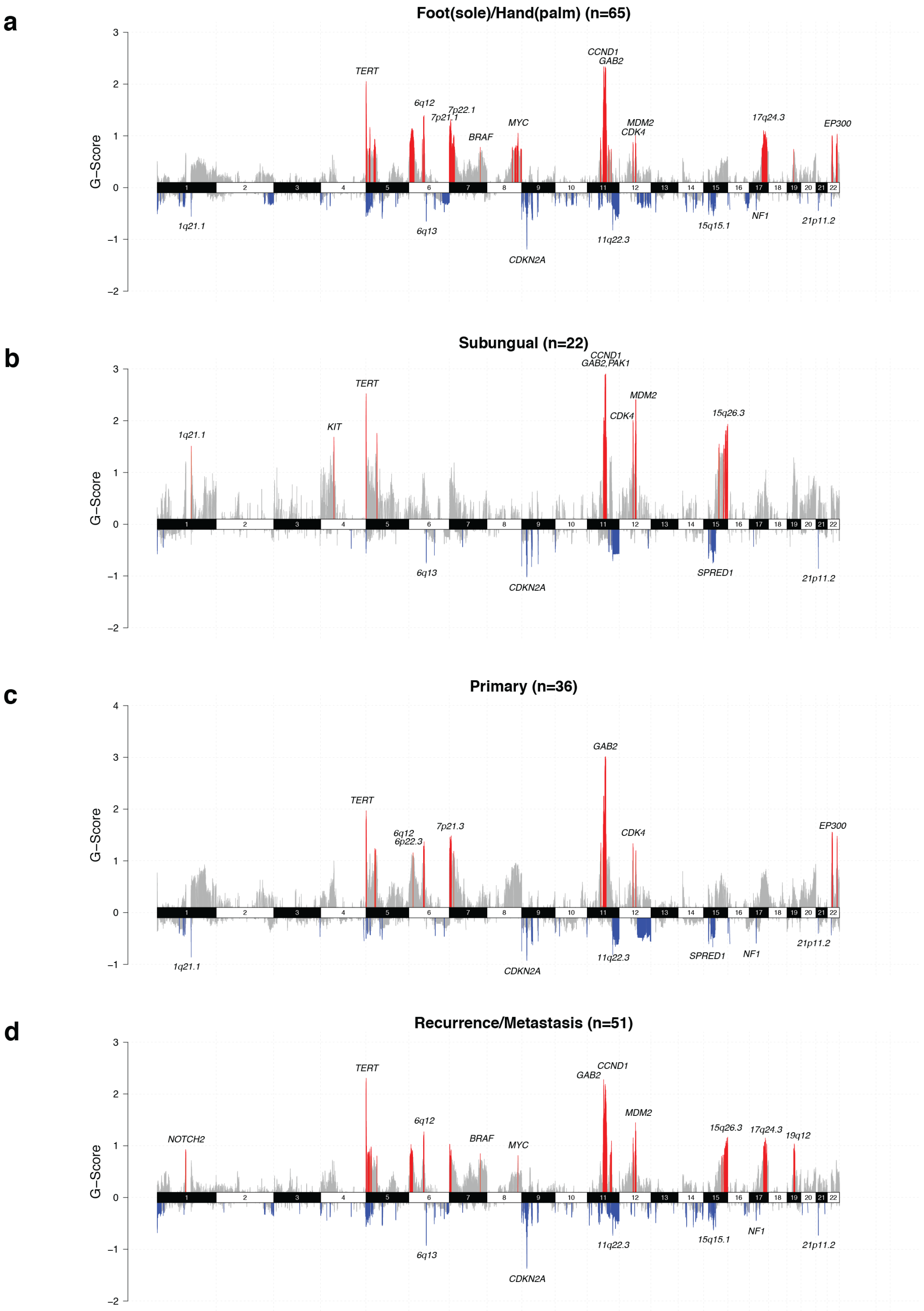

Supplementary Figure 8: GISTIC analysis in tumor subgroups. Focal regions of recurrent amplification (red) and deletion (blue) as identified by GISTIC2 in tumor subgroups. Genes and chromosomal cytobands of interest are annotated in the plot. **a**, Sole of the foot or palm of the hand primary tumor site, **b,** Subungual primary tumor site, **c,** Primary tumors, **d,** Recurrence and metastasis tumors.

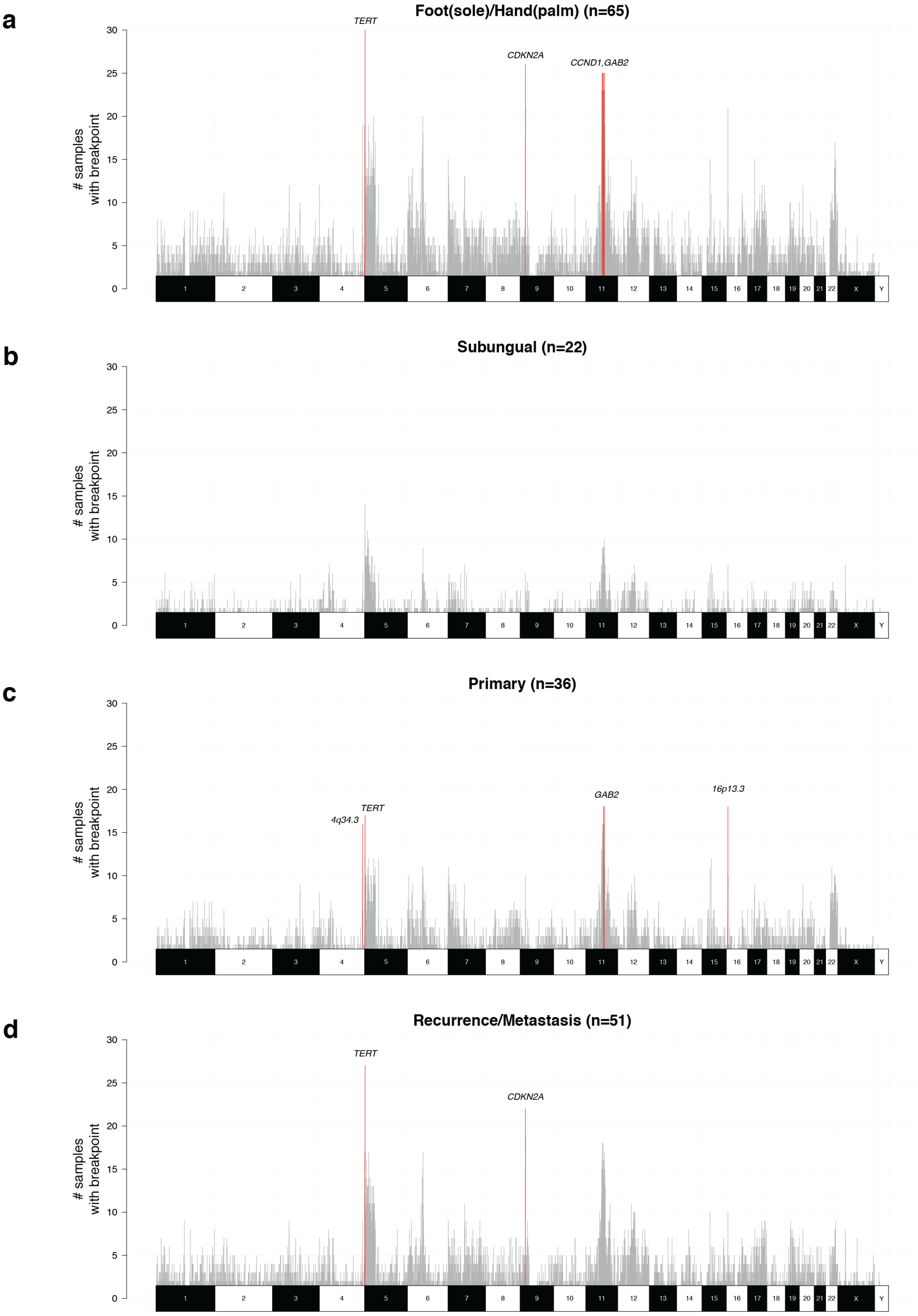

Supplementary Figure 9: Regions of recurrent rearrangement breakpoints in tumor subgroups. Regions of recurrent rearrangement breakpoints as identified by RETREAD in tumor subgroups. The plot shows 1Mb windows that have one or more rearrangements. Bars in gray represent windows that are not significant (q>0.2), and red bars indicate regions that are significantly enriched (q<0.2). **a**, Sole of the foot or palm of the hand primary tumor site. **b,** Subungual primary tumor site. **c,** Primary tumors. **d,** Recurrence and metastasis tumors.

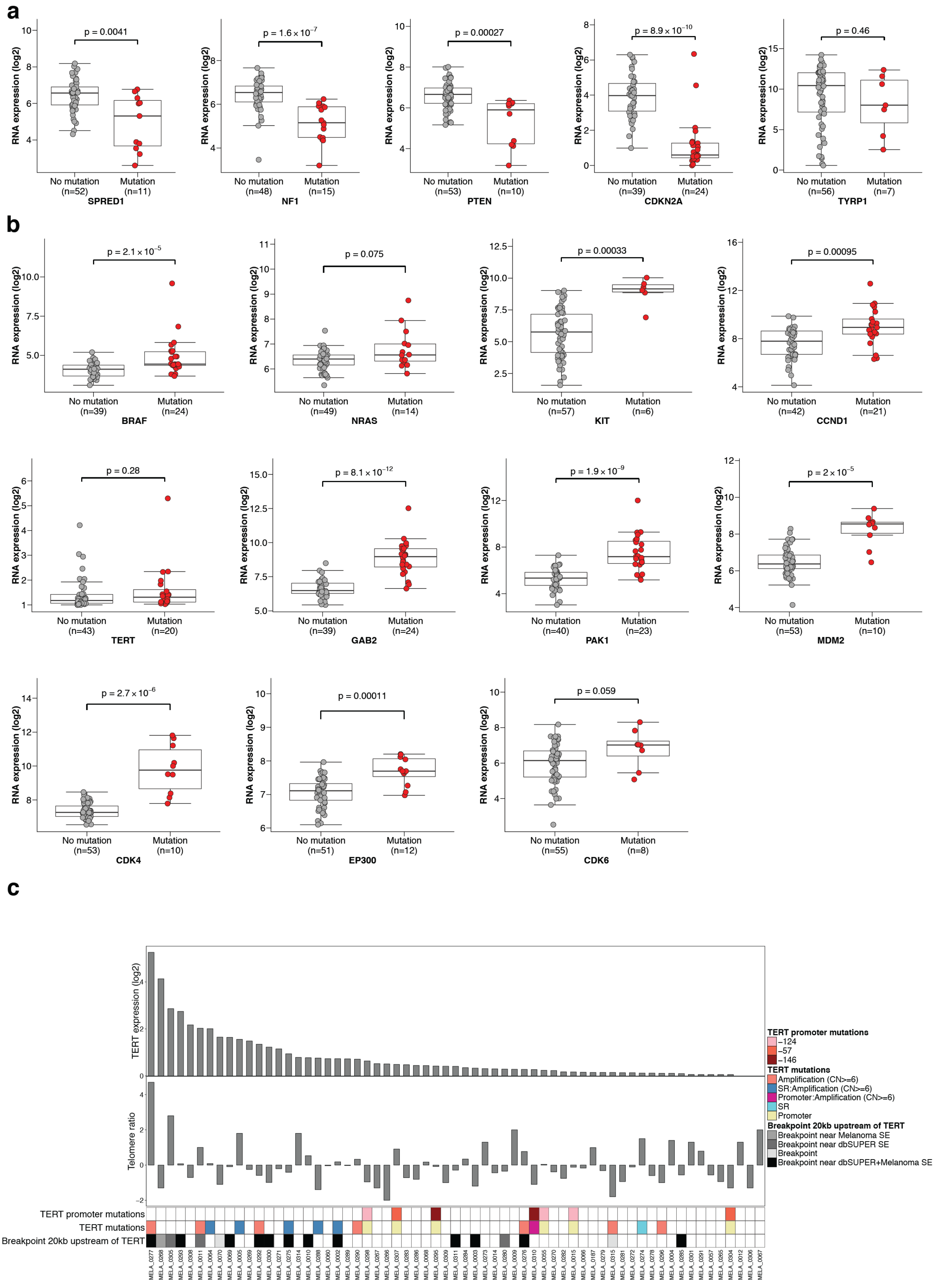
a
b
c
RNA expression (log2)
No mutation
Mutation
p = 0.0041
(n=52) (n=11)
SPRED1
p = 1.6 × 10−7
(n=48) (n=15)
NF1
p = 0.00027
(n=53) (n=10)
PTEN
p = 8.9 × 10−10
(n=39) (n=24)
CDKN2A
p = 0.46
(n=56) (n=7)
TYRP1
p = 2.1 × 10−5
(n=39) (n=24)
BRAF
p = 0.075
(n=49) (n=14)
NRAS
p = 0.00033
(n=57) (n=6)
KIT
p = 0.00095
(n=42) (n=21)
CCND1
p = 0.28
(n=43) (n=20)
TERT
p = 8.1 × 10−12
(n=39) (n=24)
GAB2
p = 1.9 × 10−9
(n=40) (n=23)
PAK1
p = 2 × 10−5
(n=53) (n=10)
MDM2
p = 2.7 × 10−6
(n=53) (n=10)
CDK4
p = 0.00011
(n=51) (n=12)
EP300
p = 0.059
(n=55) (n=8)
CDK6
TERT expression (log2)
Telomere ratio
TERT promoter mutations
TERT mutations
Breakpoint 20kb upstream of TERT
TERT promoter mutations
−124
−57
−146
TERT mutations
Amplification (CN>=6)
SR:Amplification (CN>=6)
Promoter:Amplification (CN>=6)
SR
Promoter
Breakpoint 20kb upstream of TERT
Breakpoint near Melanoma SE
Breakpoint near dbSUPER SE
Breakpoint
Breakpoint near dbSUPER+Melanoma SE

Supplementary Figure 10: Correlation of gene mutations with expression. a, Box plots of gene expression in tumors with and without mutations that could potentially lead to decreased expression (SV, Homozygous deletion - copy number 0, SNV/indel) (Mann-Whitney U test). **b**, Expression of genes with and without mostly putatively activating mutations (amplification, SNV/indel mutations) (Mann-Whitney U test). In each box plot, the box boundaries show the first to third quartiles, the median is the centre line and the whiskers represent 1.5 times the inter-quartile range. **c**, *TERT* expression, telomere ratio (log2 tumor telomere length/matched normal telomere length), *TERT* mutations and rearrangement breakpoints upstream of *TERT*. Upstream breakpoints are coloured based on whether or not they are close (within 100kb) to a super enhancer. All gene expression values are shown in log2(TMM normalized RNA-seq values+1).

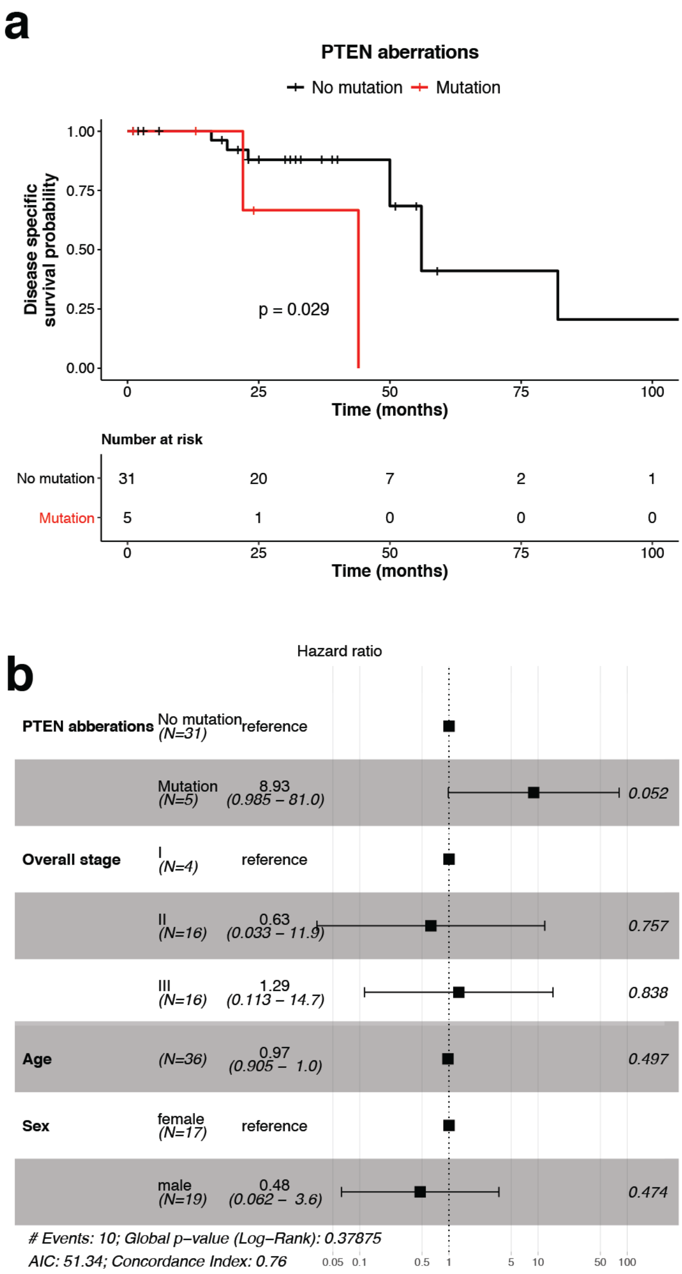

Supplementary Figure 11: Survival in primary acral melanomas with and without *PTEN* aberrations. a, Kaplan-Meier plot of melanoma-specific survival with log-rank test in primary tumors with or without *PTEN* aberrations. **b**, Forest plot for a multivariable Cox survival model based on *PTEN* aberrations, overall stage, patient age at diagnosis of primary and sex).

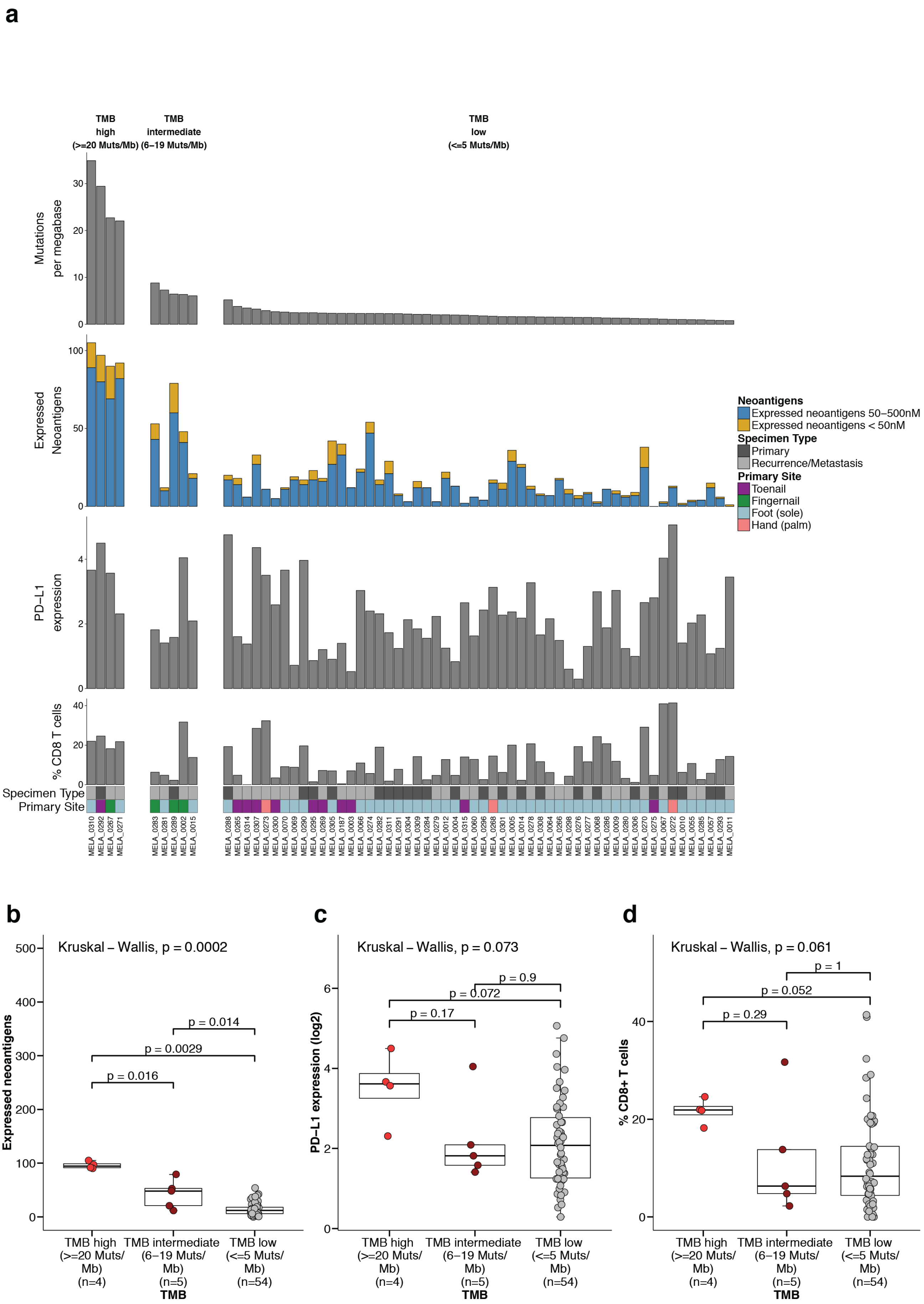

a
TMB high (>=20 Muts/Mb)
TMB intermediate (6–19 Muts/Mb)
TMB low (<=5 Muts/Mb)
Mutations per megabase
Expressed Neoantigens
PD−L1 expression
% CD8 T cells
Specimen Type
Primary Site
Neoantigens
Expressed neoantigens 50–500nM
Expressed neoantigens < 50nM
Specimen Type
Primary
Recurrence/Metastasis
Primary Site
Toenail
Fingernail
Foot (sole)
Hand (palm)
b
Kruskal – Wallis, p = 0.0002
Expressed neoantigens
p = 0.016
p = 0.0029
p = 0.014
c
Kruskal – Wallis, p = 0.073
PD−L1 expression (log2)
p = 0.17
p = 0.072
p = 0.9
d
Kruskal – Wallis, p = 0.061
% CD8+ T cells
p = 0.29
p = 0.052
p = 1
TMB high (>=20 Muts/ Mb) (n=4)
TMB intermediate (6−19 Muts/ Mb) (n=5)
TMB low (<=5 Muts/ Mb) (n=54)
TMB

Supplementary Figure 12: Neoantigens and the tumor microenvironment. a, From top to bottom: tumor mutational burden (TMB) in mutations per megabase; number of expressed neoantigens with predicted HLA binding affinity of < 500 nM; PD-L1 (*CD274*) expression shown as log2(TMM normalized RNA-seq values+1); percent of CD8 positive T cells estimated by immune cell deconvolution using CIBERSORT; specimen type group and primary site. Each barchart represents data from n=63 tumors, where each bar represents the values for a single tumor. Tumors are divided into TMB high (≥20 mutations per megabase, n=4), TMB intermediate (6-19 mutations/Mb, n=5) and TMB low (1-5 mutations/Mb, n=54). **b-d,** Box plots comparing tumors with differing TMB. In each box plot, the box boundaries show the first to third quartiles, the median is the centre line and the whiskers represent 1.5 times the inter-quartile range. The overall test for significance is a Kruskal-Wallis test and p-values for pairwise comparisons represent Mann-Whitney U tests with adjustment for multiple test correction by FDR. **b**, Expressed neoantigens **c**, PD-L1 expression (log2 TMM normalized RNA-seq counts+1) **d**, Percent of CD8 positive T cells estimated by immune cell deconvolution using CIBERSORT.
