# Supplementary material for: Whole-genome sequencing of acral melanoma reveals genomic complexity and diversity
Source: Nat Commun. 2020 Oct 16;11:5259. doi: 10.1038/s41467-020-18988-3 (PMC7567804; doi:10.1038/s41467-020-18988-3)
Supplement: Supplementary file 3 — Description of Additional Supplementary Files [file 41467_2020_18988_MOESM3_ESM.pdf]

Description of Additional Supplementary Files

File Name: Supplementary Data 1
Description: Clinical, sequencing and genomic data per patient.

File Name: Supplementary Data 2
Description: Cohort summary of clinical parameters

File Name: Supplementary Data 3
Description: Coding mutations

File Name: Supplementary Data 4
Description: Significantly mutated genes

File Name: Supplementary Data 5
Description: Predicted intergene fusions
