# Supplementary material for: Whole-genome sequencing of acral melanoma reveals genomic complexity and diversity
Source: Nat Commun. 2020 Oct 16;11:5259. doi: 10.1038/s41467-020-18988-3 (PMC7567804; doi:10.1038/s41467-020-18988-3)
Supplement: Supplementary file 9 — Reporting Summary [file 41467_2020_18988_MOESM9_ESM.pdf]

nature research

Corresponding author(s): Felicity Newell

Last updated by author(s): Sep 14, 2020

Reporting Summary

Nature Research wishes to improve the reproducibility of the work that we publish. This form provides structure for consistency and transparency in reporting. For further information on Nature Research policies, see our Editorial Policies and the Editorial Policy Checklist.

Statistics

For all statistical analyses, confirm that the following items are present in the figure legend, table legend, main text, or Methods section.

n/a	Confirmed	
☐	☒	The exact sample size (*n*) for each experimental group/condition, given as a discrete number and unit of measurement
☐	☒	A statement on whether measurements were taken from distinct samples or whether the same sample was measured repeatedly
☐	☒	The statistical test(s) used AND whether they are one- or two-sided<br>*Only common tests should be described solely by name; describe more complex techniques in the Methods section.*
☒	☐	A description of all covariates tested
☒	☐	A description of any assumptions or corrections, such as tests of normality and adjustment for multiple comparisons
☐	☒	A full description of the statistical parameters including central tendency (e.g. means) or other basic estimates (e.g. regression coefficient) AND variation (e.g. standard deviation) or associated estimates of uncertainty (e.g. confidence intervals)
☐	☒	For null hypothesis testing, the test statistic (e.g. *F*, *t*, *r*) with confidence intervals, effect sizes, degrees of freedom and *P* value noted<br>*Give P values as exact values whenever suitable.*
☒	☐	For Bayesian analysis, information on the choice of priors and Markov chain Monte Carlo settings
☒	☐	For hierarchical and complex designs, identification of the appropriate level for tests and full reporting of outcomes
☐	☒	Estimates of effect sizes (e.g. Cohen's *d*, Pearson's *r*), indicating how they were calculated

Our web collection on statistics for biologists contains articles on many of the points above.

Software and code

Policy information about availability of computer code

Data collection	No code was used for data collection
Data analysis	Adamajava: https://github.com/AdamaJava, qSNP (version 2.0), GATK HaplotypeCaller (version 3.3-0), SnpEff (version 4.0e build 2014-09-13),Cutadapt (version 1.9, version 1.11), BWA-MEM (version 0.7.12),SAMtools (version 1.1),Picard MarkDuplicates (version 1.129), qCoverage (version 0.7pre), plink (version 1.90b6.8), plinkQC R package (0.2.0), R (v3.4.4), OncodriveFML (v2.3.0), dNdScv R package (v0.0.1.0), 20/20+ (v1.1.3), ascatNgs (v4.0.1), qSV (v0.3), GISTIC (v2.0.22), qMotif (v1.2), Matlab (version R2016a), MutSigCV (v1.4), RETREAD R Package (version v 0.1.0), SigProfiler (v2.1.5.7), RNA-SeQC (version 1.1.8), RSEM (version 1.2.30), edgeR R package (3.24.3), STAR (2.5.2a), CIBERSORT (https://cibersort.stanford.edu, v1.06), Polysolver (v1.0), Optitype (v1.3.1), NMF R package (0.20.6), deconstructSigs R package (v1.8.0), pVAC-Seq (v4.0.10), NetMHCpan (v4.0), VEP (v86), CADD v1.0

For manuscripts utilizing custom algorithms or software that are central to the research but not yet described in published literature, software must be made available to editors and reviewers. We strongly encourage code deposition in a community repository (e.g. GitHub). See the Nature Research guidelines for submitting code & software for further information.

Data

Policy information about availability of data

All manuscripts must include a data availability statement. This statement should provide the following information, where applicable:

- Accession codes, unique identifiers, or web links for publicly available datasets
- A list of figures that have associated raw data
- A description of any restrictions on data availability

Sequence data that support the findings of this study have been deposited in the European Genome-phenome Archive (EGA) and are available under study accession EGAS00001001552 (https://www.ebi.ac.uk/ega/studies/EGAS00001001552) and dataset accession EGAD00001005500 (https://www.ebi.ac.uk/ega/

datasets/EGAD00001005500). Data for 1000 genomes in plink2 format are available at: https://www.cog-genomics.org/plink/2.0/resources#1kg_phase. Databases of super-enhancers are available at https://asntech.org/dbsuper/ (DBSuper database) and http://www.licpathway.net/sedb/ (SEdb database). All other data are available in the article, Supplementary Information or available from the authors upon reasonable request.

Field-specific reporting

Please select the one below that is the best fit for your research. If you are not sure, read the appropriate sections before making your selection.

[x] Life sciences [ ] Behavioural & social sciences [ ] Ecological, evolutionary & environmental sciences

For a reference copy of the document with all sections, see nature.com/documents/nr-reporting-summary-flat.pdf

Life sciences study design

All studies must disclose on these points even when the disclosure is negative.

Sample size	We sequenced 87 tumors samples using whole genome analysis. As acral melanoma is a rarer subtype of melanoma, sample size was limited by the availability of appropriate samples. We recruited as many patients as possible. As this is the largest study to date of whole genome sequenced acral melanoma samples and more than 50 more than the previous largest study, we considered this sample number sufficient.
Data exclusions	Exclusion criteria were not pre-established. Of the 87 acral tumors described in this study, no data were excluded from the analyses unless the relevant clinical data was not available for this patient. Samples where data was not available for that variable were excluded from statistical comparisons where no clinical data was available for that parameter (These samples are listed as "Not available" for that parameter is Supplementary Data 1). Associations of melanoma specific survival with SPRED1 mutation, PTEN mutation and presence of complex chromosomes, associations of tumor thickness, tumor T Classification, and the presence of ulceration with TCGA molecular subtypes and number of rearrangements, were limited to those samples had such data available (as listed in Supplementary Data 1). Analysis of associations with RNA expression was confined to those 63 samples that had matching RNAseq data available - this included associations of gene expression with mutations, expression of PD-L1, TERT and immune cell deconvolution by CIBERSORT.
Replication	Each sample was sequenced once. It is standard practice to sequence each sample once for whole-genome sequencing and it is not typical to sequence the same samples multiple times, this is a cohort study and all patients represent biological replicates. This study represents the largest cohort of a rare subtype of melanoma, and this was a descriptive study, not an experimental study, therefore replication in another dataset is not possible at this time. The variant calling pipelines used in this study have been previously validated and used in a number of other published genomic sequencing studies (including Newell et al, 2019, https://doi.org/10.1038/s41467-019-11107-x; Hayward et al, 2019 https://doi.org/10.1038/nature22071).
Randomization	Randomization was not applicable since this study comprised whole-genome sequencing of an unselected series of tumours.
Blinding	No blinding was undertaken - this was a descriptive study, not an experimental study.

Reporting for specific materials, systems and methods

We require information from authors about some types of materials, experimental systems and methods used in many studies. Here, indicate whether each material, system or method listed is relevant to your study. If you are not sure if a list item applies to your research, read the appropriate section before selecting a response.

Materials & experimental systems

n/a	Involved in the study	
[x]	[ ]	Antibodies
[x]	[ ]	Eukaryotic cell lines
[x]	[ ]	Palaeontology and archaeology
[x]	[ ]	Animals and other organisms
[ ]	[x]	Human research participants
[x]	[ ]	Clinical data
[x]	[ ]	Dual use research of concern

Methods

n/a	Involved in the study	
[x]	[ ]	ChIP-seq
[x]	[ ]	Flow cytometry
[x]	[ ]	MRI-based neuroimaging

Human research participants

Policy information about studies involving human research participants

Population characteristics	Clinic-based series of acral melanoma patients. Patients had a mean age of 68 and 52% were female.
Recruitment	Fresh-frozen tissue and matched normal germline (blood) samples were obtained from the biospecimen bank of Melanoma Institute Australia (MIA) (n=83), QIMR Berghofer Medical Research Institute (n=1), University of Colorado (n=3) and University of Zurich (n=1). AM patients suitable for surgery and with sufficient fresh-frozen tumour remaining after pathology requirements for whole-genome sequencing were used. We did not notice any factors that correlated with patients' willingness to donate tissue. We did not notice any factors that correlated with patients' willingness to donate tissue.
Ethics oversight	All samples were accrued prospectively with written informed patient consent. The study protocol was approved by the Sydney Local Health District Ethics Committee (Protocol No X15-0454 (prev X11-0289) & HREC/11/RPAH/444 and Protocol No X17-0312 (prev X11-0023) & HREC/11/RPAH/32) and cases were approved by institutional ethics committees of Melanoma Institute of Australia, QIMR Berghofer Medical Research Institute (HREC approval P452 & P2274), University of Colorado and University of Zurich.

Note that full information on the approval of the study protocol must also be provided in the manuscript.
